# Identification of Fusion Genes and Targets for Genetically Matched Therapies in a Large Cohort of Salivary Gland Cancer Patients

**DOI:** 10.3390/cancers14174156

**Published:** 2022-08-27

**Authors:** Gerben Lassche, Sjoerd van Helvert, Astrid Eijkelenboom, Martijn J. H. Tjan, Erik A. M. Jansen, Patricia H. J. van Cleef, Gerald W. Verhaegh, Eveline J. Kamping, Katrien Grünberg, Adriana C. H. van Engen-van Grunsven, Marjolijn J. L. Ligtenberg, Carla M. L. van Herpen

**Affiliations:** 1 Department of Medical Oncology, Radboud Institute for Health Sciences, Radboud university medical center, 6525 GA Nijmegen, The Netherlands; 2 Department of Pathology, Radboud university medical center, 6525 GA Nijmegen, The Netherlands; 3 Department of Human Genetics, Radboud university medical center, 6525 GA Nijmegen, The Netherlands; 4 Department of Urology, Radboud Institute for Molecular Life Sciences, Radboud university medical center, 6525 GA Nijmegen, The Netherlands

**Keywords:** salivary gland neoplasms, gene fusion, actionable aberrations, NTRK genes: high throughput nucleotide sequencing

## Abstract

**Simple Summary:**

Salivary gland cancer (SGC) is a rare and heterogeneous cancer for which limited treatment options are available in the palliative treatment setting. Characterization of the SGC genetic landscape to identify actionable aberrations is therefore important. This research aimed to comprehensively assess the prevalence of various types of actionable aberrations, including gene fusions, in a large cohort of patients with different SGC subtypes. The combined approach using RNA- and DNA-based targeted next-generation sequencing panels revealed the presence of gene fusions in half of the cases, including several fusions not previously described in SGC. Targets for genetically matched therapies were identified in 28.3–81.8% of cases, depending on the SGC subtype (overall 53.7% of the cases). This highlights the potential of molecular diagnostics to select systemic treatment in SGC.

**Abstract:**

**Introduction:** Salivary gland cancer (SGC) is a rare cancer for which systemic treatment options are limited. Therefore, it is important to characterize its genetic landscape in search for actionable aberrations, such as *NTRK* gene fusions. This research aimed to identify these actionable aberrations by combining NGS-based analysis of RNA (gene fusions) and DNA (single and multiple nucleotide variants, copy number variants, microsatellite instability and tumor mutational burden) in a large cohort of SGC patients. **Methods:** RNA and DNA were extracted from archival tissue of 121 patients with various SGC subtypes. Gene fusion analysis was performed using a customized RNA-based targeted NGS panel. DNA was sequenced using a targeted NGS panel encompassing 523 cancer-related genes. Cross-validation of NGS-based *NTRK* fusion detection and pan-TRK immunohistochemistry (IHC) was performed. **Results:** Fusion transcripts were detected in 50% of the cases and included both known (*MYB-NFIB, MYBL1-NFIB, CRTC1-MAML2*) and previously unknown fusions (including transcripts involving *RET, BRAF* or *RAD51B*). Only one *NTRK* fusion transcript was detected, in a secretory carcinoma case. Pan-TRK IHC (clone EPR17341) was false positive in 74% of cases. The proportion of patients with targets for genetically matched therapies differed among subtypes (salivary duct carcinoma: 82%, adenoid cystic carcinoma 28%, mucoepidermoid carcinoma 50%, acinic cell carcinoma 33%). Actionable aberrations were most often located in *PIK3CA* (*n* = 18, 15%), *ERBB2* (*n* = 15, 12%), *HRAS* and *NOTCH1* (both *n* = 9, 7%). **Conclusions:** Actionable genetic aberrations were seen in 53.7% of all SGC cases on the RNA and DNA level, with varying percentages between subtypes.

## 1. Introduction

Precision medicine has gained great momentum in clinical oncology practice over the past decade. This is highlighted by the rapidly increasing number of basket trials being performed, in which cancer patients are treated with therapeutic interventions targeting specific aberrations present in the patients’ tumor [1]. Recent reports of such basket trials indicate that tumor responses can be elicited in patient groups lacking other standard treatment options [2,3]. Hence, it is a promising approach to treat patients based on specific genetic aberrations which drive the tumor, especially for treatment of patients suffering from rare cancers, because it takes advantage of knowledge obtained in more common malignancies. Moreover, in rare cancers, the conventional route of drug registration is often hampered by difficulties in performing phase III trials.

Salivary gland cancer (SGC) is a rare cancer for which limited treatment options are available in the palliative treatment setting [4]. Adding to the complexity of studying SGC is its subdivision into 22 different subtypes that highly differ in clinicopathological characteristics and genetic hallmarks [4,5,6]. This merits recognition of the different subtypes as separate entities and treating them as such, but also emphasizes the importance of characterizing the molecular landscape to identify potential actionable genetic aberrations.

A common feature in the molecular landscape of SGC is the presence of gene fusions, which are believed to be dominant drivers of cancer progression [7,8]. Chromosomal rearrangements that have previously been identified in SGC are *MYB-* or *MYBL1-NFIB* gene fusions in adenoid cystic carcinoma (AdCC), *CRTC1-* or *CTRC3-MAML2* gene fusions in mucoepidermoid carcinoma (MEC), *PLAG1* or *HMGA2* gene fusions in carcinoma ex pleomorphic adenoma (CXPA) and *ETV6-NTRK3* gene fusions in secretory carcinoma [8,9,10,11,12,13,14]. *NTRK* gene fusions are of particular interest due to recent registration of targeted therapies for *NTRK* fusion-positive cancers. *NTRK* rearrangements are found in a wide variety of cancer types and they can result in the expression of ligand-independent and/or constitutive active oncogenic fusion proteins [15]. The resulting activated downstream signaling is believed to be a strong driver for these cancers, indicated by impressive response rates seen in *NTRK* gene fusion-positive cancer patients after treatment with selective TRK inhibitors, such as larotrectinib (71% response rate, with a median duration of response 35 months) and entrectinib (57% response rate with a median duration of response of 10 months) [16,17].

Various types of genetic aberrations are actionable with matched therapies. It is therefore pivotal to also test for single and multiple nucleotide variants and copy number variants in SGC. This research aims to comprehensively assess the prevalence of actionable aberrations, including gene fusions, in a large cohort of SGC patients. An RNA-based targeted next-generation sequencing (NGS) panel was used for gene fusion detection and targeted DNA-based NGS panel analysis was used to detect single and multiple nucleotide variants, copy number variants, tumor mutational burden and microsatellite instability in 121 SGCs. The combined approach revealed the presence of gene fusions in half of the cases, including several fusions not previously described in SGC, and the presence of targets for genetically matched therapies in 28.3–81.8% of cases, depending on the SGC subtype.

## 2. Methods

### 2.1. Patient Selection and Material Acquisition

Patients participating in the Radboud university medical center Biobank for SGC were included in this study. This cohort was supplemented with patients visiting the outpatient clinics of the departments of otorhinolaryngology, maxillofacial surgery, or medical oncology of the tertiary referral center of Radboud university medical center, who were suffering from, AdCC, salivary duct carcinoma (SDC), MEC or acinic cell carcinoma (AciCC). Formalin-fixed paraffin-embedded (FFPE) tissue that was not older than five years had to be available. This study was approved by the institutional review board and if possible, patients provided written informed consent (file numbers 2017-3679 and 2019-5476). FFPE material of these patients was retrieved from pathological archives, partially by the Nationwide Network and Registry of Histo- and Cytopathology in the Netherlands (PALGA) [18]. Clinicopathological characteristics were retrospectively collected from the medical records.

### 2.2. DNA and RNA Extraction

DNA and RNA were extracted from 6 µm FFPE sections, 5–10 sections per case. Adjacent 4 µm sections were stained with hematoxylin and eosin (HE) for estimation of tumor cell percentage and annotation of the tissue area containing tumor cells. The annotated tissue was manually macrodissected from the 6 µm sections. RNA was isolated using the Reliaprep FFPE Total RNA Miniprep system (Promega, Madison, WI, USA) according to manufacturers’ protocol. Genomic DNA was extracted using Chelex-100 and 400 μg proteinase K as previously described [19]. Nucleic acid concentrations were measured using the Qubit dsDNA Broad Range and RNA HS kits (Thermo Fisher Scientific, Waltham, MA, USA).

### 2.3. Detection of RNA Gene Fusion Transcripts

Anchored multiplex PCR technology was used to detect RNA gene fusion transcripts. Up to 250 ng total RNA was used for preparation of cDNA. Open-ended target-enriched NGS libraries were subsequently prepared using the FusionPlex^®^ kit according to the manufacturer’s instructions (Invitae, San Francisco, CA, USA). A custom-designed targeted gene panel was used (Radboudv1), which includes 56 genes relevant for, but not limited to, differential diagnosis of SGC and therapeutic targeting (Supplementary Table S1). Pooled FusionPlex^®^ libraries were combined with TSO500 libraries for sequencing. Demultiplexing was performed using an in-house bioinformatic workflow and data were thereafter analyzed using Archer Analysis software (ArcherDX, Boulder, CO, USA) version 6.2.7. Analysis QC was based on the percentage of reads mapped on RNA. Analyses with ≥50% RNA reads were classified as ‘good quality’, 20–50% as ‘mediocre’ and <20% as ‘fail’. The minimum average unique RNA start sites per gene-specific primer 2 for control genes was set at 10.

### 2.4. DNA Next Generation Sequencing

Presence of single and multiple nucleotide variants, copy number variants (CNVs), tumor mutational burden (TMB) and microsatellite instability (MSI) was assessed using the TruSight Oncology 500 panel (TSO500, Illumina, San Diego, CA, USA), which contains 523 cancer-related genes with a total genomic content of 1.94 Mb (Supplementary Table S2). Preparation of NGS libraries was performed according to the manufacturers’ instructions, as described before [20]. During library preparation, unique molecular identifier ligation and two-step hybridization capture-based target enrichment was used, allowing sensitive detection of mutations. Sequencing was performed using a NextSeq500 (Illumina, San Diego, CA, USA) with a high output cassette.

NGS data (TSO500) are available in the European Genome-Phenome Archive after reasonable request (Study ID EGAS00001006232).

### 2.5. Pan-TRK Immunohistochemistry

TRK expression was detected in FFPE tissue sections with pan-TRK immunohistochemistry (IHC). The rabbit monoclonal antibody EPR17341 (Abcam, Cambridge, MA, USA), targeting a conserved epitope on the TRKA, TRKB and TRKC proteins, was used on a semi-automatic Labvision immunostainer 480 or 360 (Thermo Fisher Scientific, Waltham, MA, USA) in a dilution of 1:25. Detection was performed using EnVision FLEX High Ph, HRP rabbit/mouse (DAKO Agilent, Santa Clara, CA, USA). Healthy appendix tissue was used as a positive control. Scoring was performed by an expert SGC pathologist (AvEvG). Samples were divided into four categories after comparing to the negative and positive control: negative, weak/dubious, moderate and positive. In case of tissue shortage, priority was given to NGS over IHC analysis.

### 2.6. NTRK1, NTRK2 and NTRK3 Fluorescence In Situ Hybridization

Fluorescence in situ hybridization (FISH) was performed for cases with discordant results in pan-TRK IHC and gene fusion analysis (only when RNA-NGS QC was mediocre or fail). Dual-color break-apart probes targeting *NTRK1* (z-2167-200), *NTRK2* (z-2205-200) and *NTRK3* (z-2206-200) were used according to manufacturer’s instruction (ZytoVision, Bremerhaven, Germany). Fifty nuclei were scored by two independent researchers, using a Leica DMRBE (Leica, Wetzlar, Germany) fluorescence microscope. Samples were considered positive if >50% of nuclei scored positive (1 yellow, 1 green and 1 red signal), and dubious if 10–50% of nuclei scored positive. Dubious results were considered positive if >15% of nuclei scored positive after scoring by the second researcher.

### 2.7. TSO500 Data Analysis

Sequencing data of the TSO500 panel was processed using the TSO500 Local App Version 2.0.0.70 (Illumina, San Diego, CA, USA). GRCh37/hg19 was used as a reference genome. This pipeline calls variants along with their allele frequencies and reports total and non-synonymous tumor mutational burden (TMB) and the percentage of microsatellite instable sites (MSI). Non-synonymous TMB values are further used in this study and referred to as TMB. Cases were considered MSI high if the percentage of unstable sites was ≥25% and uncertain with 10–25% unstable sites. Called variants were filtered by excluding synonymous variants, variants in non-coding regions outside of splice sites (including the 3′ and 5′ untranslated region (UTR)) and variants that have a prevalence >0.1% in the general population (assessed by crosschecking the variant in the Exome Aggregation Consortium database (version 0.2). Variants were assessed in a subset of genes (Supplementary Table S3), that were mainly selected because mutations in these genes could lead to treatment with currently registered drugs or in basket trials (such as the Drug Rediscovery protocol, NCT02925234). Single and multiple nucleotide somatic variants were rated in a five-tier classification of pathogenicity: 1. benign (single nucleotide polymorphisms (SNPs)), 2. likely benign, 3. variant of unknown/uncertain significance (VUS), 4. likely pathogenic or 5. pathogenic, as recommended by the American College of Medical Genetics and Genomics and the Association for Molecular Pathology [21]. Determination of pathogenicity was performed by a clinical scientist in molecular pathology (SvH). Variants classified as likely pathogenic and pathogenic were included for further analysis. Copy number variants were determined by calculating the relative coverage per gene, followed by comparison to coverage data obtained from healthy tissues, as previously reported [22]. In general, a minimum of 50% tumor cells was required for analysis of copy number losses. Bi-allelic deletions were assessed for all tumor suppressor genes in the virtual panel, except for *TP53*. For the same tumor suppressor genes, loss of heterozygosity (LOH) was assessed only when a (likely) pathogenic mutation was detected. Actionability of the genes of the virtual panel is defined in Supplementary Table S3. Regarding gene fusions, activating *ABL1, ALK, BRAF, EGFR, FGFR1-3, MAML2, MET, NRG1, NTRK1-3, RET* and *ROS1* fusions were considered potentially actionable. 

### 2.8. Data Analysis

All patients for whom one or both NGS panels were performed (FusionPlex^®^ RadboudV1 or TSO500) were included in the analysis. Cases were considered *NTRK* gene fusion-positive based on results obtained from RNA NGS or FISH.

Descriptive statistics were used to summarize clinical data, analyzed using SPSS version 25.0 (IBM Crop. Armonk, NY, USA). Survival curves were constructed in Python version 3.8.8 with the Matplotlib, Pandas and Lifelines packages, using Kaplan–Meier estimates. 95%-confidence intervals (CI) for median survival were calculated using the exponential Greenwood formula. Oncoplots were created using maftools package in R version 4.1.2.

## 3. Results

### 3.1. Included Patients Comprise Diverse Subtypes of Salivary Gland Cancer

A total of 139 patients were included in this study and NGS data were acquired for 121 patients. In total 118 FusionPlex^®^ RadboudV1 and 119 TSO500 panels were performed (both panels were performed for 116 patients). The primary tumor was the tissue source in 71 cases (58.7%), a local recurrence in 7 cases (5.8%) and a metastatic site in 43 cases (35.5%). Absence of tumor material or material of insufficient quality was the reason for inability to perform NGS in the other cases.

The 121 patients included in the analysis consisted of 46 AdCC patients, 44 SDC patients, 16 MEC patients, 9 AciCC patients and 6 patients with other subtypes (one secretory carcinoma, one polymorphic adenocarcinoma (PAC), one adenocarcinoma NOS, one myoepithelial carcinoma, one epithelial/myoepithelial carcinoma and one mixed PAC/myoepithelial carcinoma).

For all 121 patients, median age at diagnosis was 57 years (range 17–90) and 47.9% were male. Most patients suffered from SGC in one of the major salivary glands (66.9%). The primary tumor was located outside the salivary glands in 9.9% of the cases (e.g., lacrimal gland or bronchus). At diagnosis, 15.7% presented with metastatic disease, and in 18.2% of cases the initial treatment intent was palliative. Of the patients treated with curative intent, 63.6% developed recurrent/metastatic disease during follow-up. During disease, 47.1% of the patients received systemic therapy (with a median of one line of systemic therapy). Table 1 lists clinical characteristics for the grouped cohort and per subtype.

**Figure 1:**
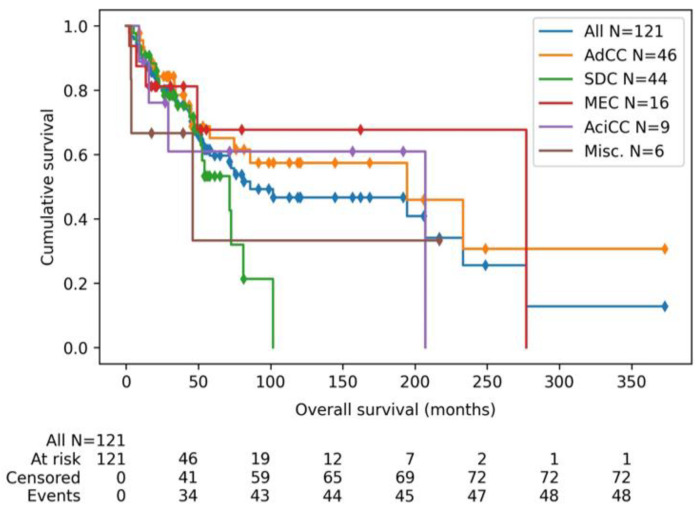
Kaplan–Meier curve of overall survival (from initial diagnosis until death), sorted per subtype and grouped for all subtypes together. All included patients are plotted, which is a mixed group regarding disease stage. Abbreviations: AdCC: adenoid cystic carcinoma, SDC: salivary duct carcinoma, MEC: mucoepidermoid carcinoma, AciCC: acinic cell carcinoma, Misc.: miscellaneous.

After median follow-up of 40 months (range 2–378) Kaplan–Meier estimates indicated a median overall survival of 86 months from initial diagnosis (95%-CI 58–233 months) for all subtypes grouped. The median overall survival of all stages in AdCC patients was 195 months (95%-CI 58-not evaluable (NE)), in SDC patients 72 months (95%-CI 47–81 months), in MEC patients 283 months (95%-CI 49–283), in AciCC patients 207 months (95%-CI 9–207) and in the miscellaneous group 46 months (3-NE) (Figure 1).

### 3.2. Half of SGC Cases Harbor Gene Fusions with Varying Incidence among Subtypes

**Figure 2:**
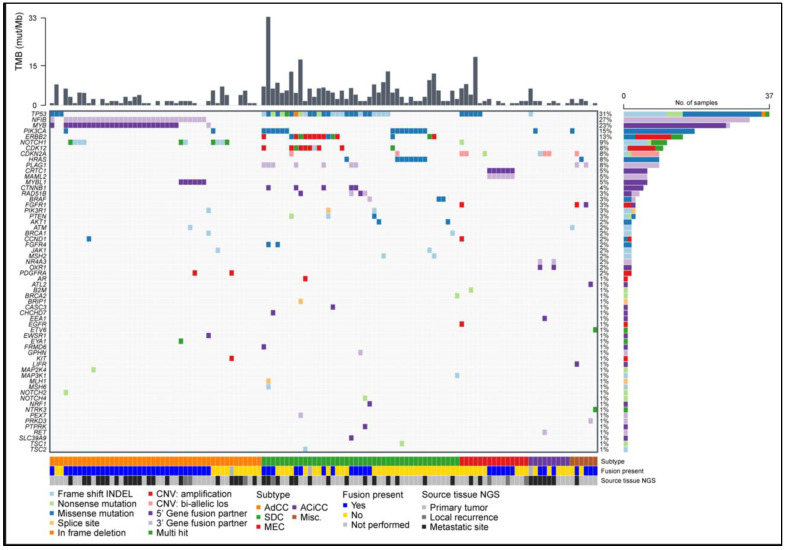
Oncoplot of all gene fusions, copy number variants and small variants that are assessed as pathogenic or likely pathogenic. AdCC: adenoid cystic carcinoma, SDC: salivary duct carcinoma, MEC: mucoepidermoid carcinoma, AciCC: acinic cell carcinoma, Misc.: miscellaneous.

To detect relevant gene fusions in this cohort of mixed subtypes, a customized NGS panel was used (FusionPlex^®^ RadboudV1). 115 of the 118 analyses were of sufficient quality. A fusion transcript was detected in 50.4% (58 out of 115) of these patients (Figure 2, Supplementary Table S4). The incidence varied per subtype, with detection of a fusion transcript in AdCC patients in 33 out of 44 (75.0%), in SDC in 12 out of 42 cases (28.6%), in MEC in 6 out of 15 (40.0%), in AciCC 3 out of 8 (37.5%) and 4 out of 6 (66.7%) in the miscellaneous group (Figure 2).

### 3.3. Frequent Detection of MYB- and MYBL1-NFIB Fusion Transcripts in AdCC

**Figure 3:**
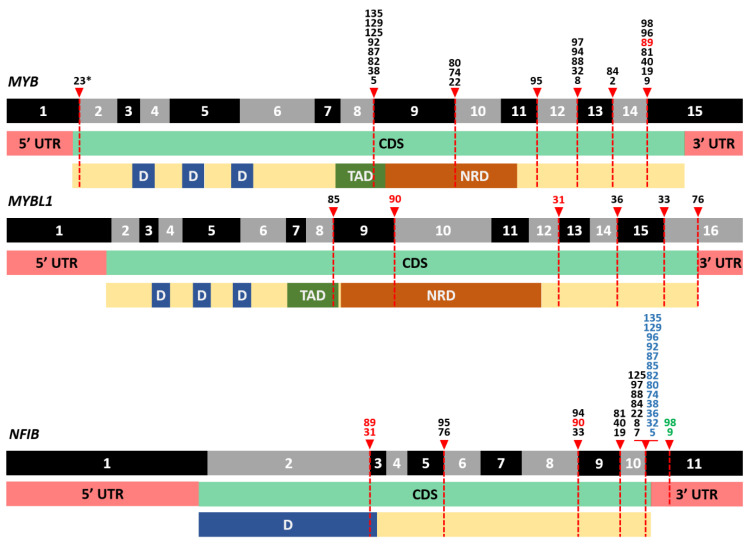
Overview of identified breakpoints in the *MYB*, *MYBL1* and *NFIB* genes and their location in functional domains of the proteins encoded by these genes. Numbers indicate unique cases. Used reference sequences are NM_005375.4 (*MYB*), NM_001080416.4 (*MYBL1*) and NM_001190737.2 (*NFIB*) and functional domains are derived from UniProt. The last exon and 3′UTR of each gene is not up to scale. In cases where more than one fusion transcript involving the same two genes was identified, the breakpoint of the dominant transcript (highest number of reads) is depicted. Red numbers: cases with an insertion between *MYB/MYBL1* and *NFIB.* Blue numbers: breakpoints mapped to exon 10 of *NFIB* transcript NM_001282787.1, which is absent in NM_001190737.2. Green numbers: cases with breaking point in 3′ UTR, lacking last amino acids of NFIB (exact breaking point not up to scale). * MYB as 3′ partner. Abbreviations: UTR: untranslated region, CDS: coding DNA sequence, D: DNA-binding.

In AdCC, all identified fusion transcripts involved *MYB* or *MYBL1,* fused to *NFIB* (*n* = 33) and fusions involving these genes were exclusive to AdCC cases. Fusions with *MYB* or *MYBL1* gene as 5′ partner and *NFIB* as 3′ partner were detected most frequently (*n* = 32, Figure 2 and Figure 3). In most of these cases (*n* = 22) one or more alternative fusion transcripts involving the same genes were detected, with different breakpoints, indicating alternative or aberrant splicing of the fusion transcripts. One in-frame *EWSR1-MYB* fusion transcript was detected, with *MYB* as 3′ partner, leaving the *MYB* coding sequence largely intact (fusion of exon 8 of *ESWR1* (NM_005243.3) and exon 2 of *MYB* (NM_005243.3)). This fusion has been described before in a myelofibrosis case and is believed to lead to upregulation of *MYB* [23].

All *MYB* and *MYBL1* fusion transcripts contained the DNA binding and (the majority of) the transactivation domain (Figure 3). Apparently, presence of these domains in the context of NFIB 3′ UTR or its downstream genomic region are sufficient to serve as a driver. The contribution of the *NFIB* coding sequence appeared negligible in most cases, as in the majority of cases only a minor part of the *NFIB* open reading frame was retained in the fusion transcripts (Figure 3).

Three AdCC cases harbored an insertion in between *MYB/MYBL1* and *NFIB*. In one case *MYBL1* exon 12 was fused to exon 2 of the *EYA1* gene (NM_000503.5, both genes are located in close proximity to each other on chromosome 8, 8q13.1 and 8q13.3, respectively) and *EYA1* exon 6 fused to *NFIB* exon 3 into a triple gene fusion. In the second case, exon 2 of *PDE7B* (NM_018945.3) was inserted between exon 14 of *MYB* and exon 3 of *NFIB,* resulting in an open reading frame. The third case contained an insertion of 51 nucleotides (aligning to chromosome 8) in between exon 9 of *MYB* and exon 9 of *NFIB,* resulting in an out-of-frame fusion transcript.

### 3.4. A Plethora of Both Known and New Fusion Transcripts was Detected in Non-AdCC Cases

Fusion transcripts involving *PLAG1* as 3′ partner, frequently seen in SGC arising from pleomorphic adenomas (CXPA), were detected in SDC (*n* = 7), myoepithelial carcinoma (*n* = 1) and a case with mixed PLGA/myoepithelial histology (9). In eight out of these nine cases earlier diagnostic pathology did reveal that these tumors did arise from pleomorphic adenomas. In the only case in which CXPA origin could not be confirmed, only biopsy material was available for diagnostic pathology, which might lead to missing the pleomorphic adenoma origin. The complete coding sequence of *PLAG1* was retained in all fusion transcripts. Presumably the *PLAG1* start codon is used, as in eight out of nine cases the start codon of the 5′ partner is not present in the fusion transcript and in the other case the start codon of the 5′ partner is followed by 2 non-coding exons of *PLAG1*. The 5′ fusion partner genes were *CTNNB1* (*n* = 5)*, CHCHD7, FGFR1, FRMD6* or *LIFR* (*n* = 1 each) (Figure 2).

*CRTC1-MAML2* fusion transcripts were detected in six cases (all in-frame). As expected, they were all detected in MEC (five low grade, one intermediate grade) [12]. In two AciCC cases an *OXR1-NR4A3* fusion transcript was identified, which contained a short 5′ sequence of *OXR1* fused to the complete coding sequence of *NR4A3*. Analogous to *PLAG1-*type translocations, this could result in a pathogenic effect due to exchange of regulatory sequences, rather than formation of a chimeric fusion protein. In line with these findings, enhancer hijacking has been shown to result in overexpression of the oncogenic *NR4A3* [24], although *OXR1* as fusion partner has not yet been described.

In four cases (all SDC) fusion transcripts involving *RAD51B* were detected, twice as 5′ and twice as 3′ fusion partner. These fusions probably lead to inactivation of *RAD51B.* It has been shown that even in the presence of a wild-type allele this can lead to homologous recombination deficiency due to haploinsufficiency [25]. In one of these cases, a second fusion transcript involving *PLAG1* was detected (Supplementary Table S4).

Regarding *NTRK* gene fusions, only one *ETV6-NTRK3* fusion transcript was detected in the secretory carcinoma case (as was the reciprocal *NTRK3-ETV6* transcript). No other fusion transcripts involving either one of the *NTRK* genes were detected.

Four other in-frame fusion transcripts were detected once: an *ATL2D-PRKD3* fusion transcript was detected in a PAC case and an *EEA1-RET* fusion transcript in an AciCC case (in-frame, kinase domains of *RET* retained). In one SDC, an in-frame *CASC3-ERBB2* fusion transcript was detected with retained *ERBB2* kinase domain. These genes are both located in close proximity on chromosome 17. This case was shown to harbor an *ERBB2* amplification in the TSO500 analysis. In an SDC case an *NRF1-BRAF* fusion transcript was detected, which has been described before in other tumor types and is believed to lead to activation of downstream MAPK signaling [26].

### 3.5. Pan-TRK Immunohistochemistry Is False Positive in the Majority of SGC Cases

To enable treatment with TRK-inhibitors in case of incurable recurrent or metastatic disease detection of *NTRK* fusions is pivotal, for which pan-TRK IHC is often used as initial screening prior to NGS. To assess performance of this screening in SGC, pan-TRK IHC was performed in 108 cases. Compared to the negative control, weak cytoplasmatic IHC positivity was observed frequently, as was positive myoepithelium in AdCC cases (Supplementary Figure S1). Out of the 108 cases, 28 were negative (25.9%) and the other cases were scored as positive (clearly positive (*n* = 11, 10.2%), weakly/dubious positive (*n* = 47, 43.5%) or moderately positive (*n* = 22, 20.4%)). Of the AdCC cases, 82.5% scored positive, in SDC cases 77.5%, in MEC cases 53.3%, in AciCC cases 37.5% and in the miscellaneous group including the secretory carcinoma case with the *ETV6-NTRK3* gene fusion all cases scored positive.

FISH was performed for 19 of 24 cases with a positive TRK-IHC and NGS data of mediocre quality. All the *NTRK1, NTRK2* and *NTRK3* FISH samples were scored negative, although two cases had a dubious *NTRK1* result (Supplementary Figure S2). Polysomy was detected frequently.

With the NGS and FISH results combined, an *NTRK* gene fusion was detected in 1 out of 118 cases (the secretory carcinoma case). Of the 80 cases that did not score negative on IHC, 79 cases were false positive, leading to an overall false positivity rate of pan-TRK IHC of 73.8% (79 out of 107). Given the overall low prevalence of *NTRK* gene fusions, sensitivity of IHC to detect *NTRK* gene fusions could not be assessed reliably.

### 3.6. High TMB and MSI Are Rare in SGC

To assess actionability with immune checkpoint inhibitors, TMB and microsatellite status were assessed. At least 1.2 megabases of coding regions were sequenced in each of the 119 TSO500 panels (46 AdCC cases, 43 SDC cases, 15 MEC cases, 9 AciCC cases and 6 miscellaneous cases). Median exon coverage ranged from 80–904 (median 308) unique reads and the median percentage of exon coverage with at least 100 unique reads was 96.6% (Supplementaryf Figure S3). 

**Figure 4:**
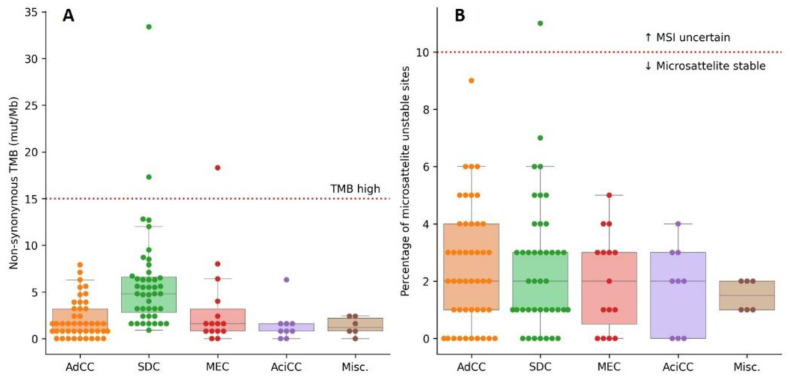
(**A**): Non-synonymous tumor mutational burden (TMB), sorted per subtype. (**B**): percentage of microsatellite instable sites (MSI), sorted per subtype. Dotted lines indicate thresholds for tumors with high TMB and with potential MSI. Abbreviations: SDC: salivary duct carcinoma, AdCC: adenoid cystic carcinoma, MEC: mucoepidermoid carcinoma, AciCC: acinic cell carcinoma, misc.: miscellaneous.

The median TMB for all subtypes grouped was 1.6 mut/Mb and ranged from 0.0–33.4 mut/Mb. The median TMB was highest in SDC cases with 4.8 mut/Mb. In AdCC median TMB was 1.6 mut/Mb, in MEC cases 1.6 mut/Mb, in AciCC cases 0.8 mut/Mb and in the miscellaneous group 1.2 mut/Mb (Figure 4A). Three tumors qualified as TMB-high, with 17.3 and 33.4 mut/Mb (SDC) and 18.3 mut/Mb (MEC).

Microsatellite status was determined by the percentage of unstable sites for MSI. Initially, no MSI was detected, with a median percentage of unstable sites in all subtypes grouped of 2% (range 0–11%, Figure 4B). One SDC case scored uncertain with 11% (12 of 106) unstable sites. In this case an *MLH1* mutation with LOH was detected and MLH1 IHC confirmed loss of nuclear MLH1 proteins. The same case also harbored the highest TMB (33.4 mut/Mb).

### 3.7. Pathogenic Small Nucleotide Variants Are Most Frequent in SDC

Single nucleotide and small insertion and/or deletion variants identified within a virtually defined panel of 61 genes (focusing on but not restricted to genes that may direct treatment decisions, Supplementary Table S3) were assessed for their pathogenicity. In total, 381 variants were assessed, of which 125 were classified as pathogenic or likely pathogenic somatic mutations, identified in 72 different cases (60.5%). These 125 (likely) pathogenic variants were located in 28 different genes (Figure 2). Mutations in *TP53, PIK3CA* and *NOTCH1* were most frequently observed. Twelve *NOTCH1* mutations were activating (i.e., truncating mutations deleting the PEST domain), as was the only *NOTCH2* mutation [27,28]. All were detected in AdCC cases. Four truncating *NOTCH1* mutations, detected in three cases (two AdCC and one MEC), were considered inactivating, as too much of the open reading frame was lost. Nevertheless, they were classified as likely pathogenic because *NOTCH1* is also described as a tumor suppressor [29].

### 3.8. Copy Number Variants Are Mostly Restricted to ERBB2 Amplifications in SDC

Gene amplifications were observed in 17 cases, mostly in SDC (*n* = 13), but also in AdCC (*n* = 2), MEC (*n* = 1) and myoepithelial carcinoma (*n* = 1) (Figure 2, Supplementary Figure S4). The most frequently amplified gene was *ERBB2* (*n* = 11), often co-occurring with amplification of the nearby gene *CDK12* (*n* = 8) (Figure 2, Supplementary Figure S4). AR amplification was seen in a primary tumor specimen (SDC) that was resected from a hormone-naïve patient, indicating that the amplification was not a result of therapy-driven resistance. Bi-allelic loss of *CDKN2A* was seen in seven cases (SDC, MEC and AciCC two cases each and one myoepithelial carcinoma case, Figure 2).

### 3.9. DNA and RNA Analysis Reveals Actionable Targets in the Majority of SGC Cases

**Figure 5:**
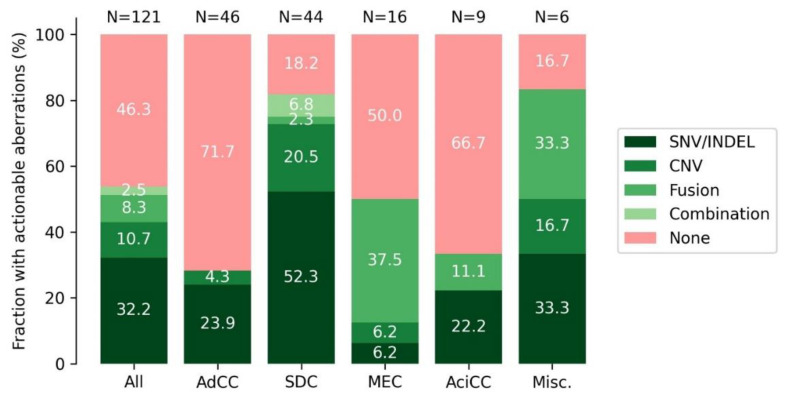
Fraction of cases with putatively actionable aberrations, split per subtype and type of aberration. The SNV/INDEL group for SDC contains one SDC case on which actionability was based solely on high TMB and one SDC case with MSI. Abbreviations: SDC: salivary duct carcinoma, AdCC: adenoid cystic carcinoma, MEC: mucoepidermoid carcinoma, AciCC: acinic cell carcinoma, misc.: miscellaneous; SNV: single nucleotide variant, INDEL: insertion/deletion; CNV: copy number variant; TMB: tumor mutational burden; MSI: microsatellite instability.

The results of the FusionPlex^®^ RadboudV1 and TSO500 were combined to estimate the fraction of SGC tumors that harbor a (potentially) actionable genetic aberration. Such aberrations (defined in Supplementary Table S3) were identified in 53.7% of all SGC cases. This varied per subtype: 28.3% for AdCC, 81.8% for SDC, 50.0% for MEC, 33.3% for AciCC and 83.3% for the miscellaneous group (Figure 5). In most subtypes the majority of actionable aberrations were single nucleotide variants or insertions/deletions, except for MEC, in which gene fusions *(MAML2)* were the most common potentially actionable aberration (Figure 5). Putatively actionable aberrations were most often located in *PIK3CA* (*n* = 18, 14.9%), *ERBB2* (*n* = 15, 12.4%), *HRAS* and *NOTCH1* (both *n* = 9, 7.4%). Actionable *ERRB2* aberrations were exclusive to SDC and actionable *NOTCH1* mutations to AdCC. *PIK3CA* and *HRAS* aberrations were most often seen in SDC (Figure 2).

## 4. Discussion

In this study we comprehensively assessed the genetic landscape of different subtypes of SGC, with a focus on gene fusions and actionable aberrations. The fraction of patients with actionable genetic tumor aberrations differs among SGC subtypes, ranging from 28.3% in AdCC to 81.8% in SDC in this cohort. Gene fusions were identified in half of all SGC cases. Except for the only secretory carcinoma case in this study (a SGC subtype known to harbor *NTRK3* fusions), no *NTRK* gene fusions were detected in other SGC subtypes [14].

As *NTRK* gene fusions are highly relevant with respect to systemic treatment options, the gene fusion analysis was focused on detection of these translocations. In two recent studies from the same group, the fractions of SGCs with *NTRK* gene fusions were 5.08% and 5.29% (13 out of 256 and 12 out of 227 cases, respectively) [30,31]. The first study, however, did not report on the SGC histological subtype, and in the second study 11 out of 12 *NTRK-*positive cases were secretory carcinomas, and one was SDC. Together with our findings, this suggests that *NTRK* gene fusions in SGC are mostly restricted to secretory carcinoma. The prevalence of *NTRK* fusions in other SGC subtypes do not seem to be higher than in other cancers (0.28% pan-cancer, 95%-confidence interval 0.22–0.35%) [30,31]. Given the low prevalence of *NTRK* fusions and the high cost of detection by NGS techniques, pan-TRK IHC is often used as a fast and inexpensive screening method. Prior studies on usefulness of pan-TRK IHC (using the same EPR17341 antibody) reported sensitivities ranging between 87.9–100% and specificities between 81.1–95.2% in all cancers, although with lower specificity for SGC (52% specificity, 88.9% sensitivity) [31,32]. We observed false positivity of pan-TRK IHC for *NTRK* gene fusion detection in 74.8% of cases, mostly due to weak cytoplasmic staining, especially in the myoepithelium of AdCC tumors. Up to approximately 100-fold higher *NTRK3* (protein alias TRKC) expression in AdCC compared to normal salivary gland tissue has been reported in the absence of activating mutations, supporting our observation [33]. This TRKC overexpression possibly leads to oncogenic downstream TRK-signaling due to autocrine production of the TRKC ligand neurotrophin-3 [33]. The consequence of this TRKC overexpression to efficacy of TRK-inhibition in AdCC patients is unknown. The sensitivity of pan-TRK IHC could be improved by more stringent scoring criteria, such as requirements that all tumor cells should score positive or that positivity should not be restricted to myoepithelial cells only. This however might lead to missing *NTRK* fusion-positive cases (i.e., reduced sensitivity) [34]. In our opinion, the results of this study and existing literature advocate for not using IHC as screening for *NTRK* fusion detection in SGC. In cases in which *NTRK* gene fusions are suspected (i.e., secretory carcinoma is in the differential diagnosis), gene fusion analysis or *NTRK3* FISH should be performed.

Gene fusions were identified in half of all cases in our cohort. The most common rearrangements were fusions between the *MYB* or *MYBL1* genes and *NFIB* in AdCC. These fusions lead to overexpression of *MYB* or *MYBL1* in a mutually exclusive way [35]. Both *MYB* and *MYBL1* encode transcription factors that exert interchangeable effects on target gene expression [36]. The DNA-binding domains and (majority of) the transactivating domain of *MYB* or *MYBL1* were preserved in all cases (Figure 2), in line with a previous report [35]. The negative regulatory domain was (partially) lost in some cases, but not in all, suggesting that loss of this domain is not solely responsible for the overexpression of *MYB* or *MYBL1.* Regarding *MYB* fusions, it has been shown that juxtaposition of super-enhancers downstream of *NFIB* near the *MYB* locus drive the *MYB* expression. MYB protein can bind to these super-enhancers, thereby creating a positive feedback loop driving AdCC [37]. In the biphasic nature of AdCC, consisting of basal myoepithelial and luminal ductal epithelial cells in AdCCs with tubular or cribriform growth patterns, *MYB* seems to drive different regulatory programs in these cell types by interplay with TP63 and NOTCH signaling [37]. Occurrence of *NOTCH* gain-of-function mutations can lead to tipping of this balance, resulting in a solid growth pattern with loss of myoepithelial cells, which is associated with markedly poorer prognoses [27,37]. Our results again emphasize the importance of *MYB/MYBL1* gene fusions in AdCC tumor biology and their specificity to AdCC.

In this study we focused on targets for genetically matched therapies by analyzing a subset of genes in the sequencing panel (Supplementary Table S3). Thereby we estimated the fraction of patients harboring a potentially actionable genetic aberration per subtype (Figure 5). This included aberrations that would allow targeted treatment in basket trials (such as the Drug Rediscovery protocol, NCT02925234) [2]. However, the true benefit of such a treatment remains inconclusive. In addition, we included some aberrations for which targeted treatment in clinical trials is probably possible in the near future. This included *MAML2* gene fusions (targetable with EGFR inhibitors) and activating *NOTCH* mutations (targetable with for instance γ-secretase inhibitors). The actionability of the presented genetic aberrations thus varies with time, as it is influenced by the availability of clinical trials and approval of new drugs. The reported fractions of patients should therefore be considered as a snapshot, highlighting the current potential treatment options for SGC. The reported fraction of 53.7% of patients with actionable aberrations, however, matches a recent study, which identified actionable aberrations in 53% of rare cancer patients by whole-genome sequencing [38]. A second recent study also matches several of our observations, including a markedly higher proportion of SDC patients with actionable aberrations compared to AdCC patients [39]. In the latter study actionable aberrations were identified in a lower percentage (27%) of the cases. This could be attributed to the use of a smaller NGS panel compared to our study, which also could not detect gene fusions. In addition, a high proportion of sequenced tumors in this study were AdCCs, which harbor the lowest amount of actionable aberrations.

A limitation of this study is that only one sample per patient was sequenced, which was the primary tumor in the majority of cases (58.7%). Possible heterogeneity between different disease sites could therefore not be assessed. In AdCC it is for instance known that the genomic landscape can differ significantly between primary tumors and recurrent/metastatic sites [40]. Over time the genomic landscape can also alter. Sequencing of paired samples located at different disease sites, sampled over time or pre- and post-treatment could overcome this and aid in assessing clinical relevance of actionable aberrations. The results of this study advocate treatment of SGC patients with genetically matched therapies, and some of the patients included in this study did indeed benefit from such treatments. Description of the different therapies that were given and the treatment outcomes are beyond the scope of this study. Nevertheless, future clinical trials using genetically matched therapies in SGC patients are warranted.

## 5. Conclusions

In conclusion, we identified previously described and novel gene fusions in half of all SGC cases, but no *NTRK* gene fusions in other subtypes than secretory carcinoma. Pan-TRK IHC false positivity is observed in 73.8% of SGC cases and is therefore not useful as initial screening for *NTRK* gene fusions in SGC. Of all SGC patients, 53.7% harbor an actionable genetic aberration, possibly leading to therapeutic options, but this highly varies across subtypes. This highlights the potential of molecular diagnostics to select systemic treatment in SGC.

## Figures and Tables

**Table 1:** Clinicopathological characteristics of the included patients. AdCC: adenoid cystic carcinoma, SDC: salivary duct carcinoma, MEC: mucoepidermoid carcinoma, AciCC: acinic cell carcinoma, misc.: miscellaneous; R/M: recurrent/metastatic.

		All (*n* = 121)	AdCC (*n* = 46)	SDC (*n* = 44)	MEC (*n* = 16)	AciCC (*n* = 9)	Misc. (*n* = 6)
	***n* (%)**						
**Age at diagnosis**							
	**Median (range)**	57 (17–90)	53 (21–83)	63 (35–90)	53 (17–72)	56 (45–71)	67 (48–79)
**Gender**							
	**Male**	58 (47.9)	15 (32.6)	28 (63.6)	8 (50)	5 (55.6)	2 (33.3)
	**Female**	63 (52.1)	31 (67.4)	16 (36.4)	8 (50)	4 (44.4)	4 (66.7)
**Location primary tumor**							
	**Major salivary gland**	81 (66.9)	21 (45.7)	42 (95.5)	6 (37.5)	9 (100.0)	3 (50.0)
	**Minor salivary gland**	28 (23.1)	16 (34.8)	1 (2.3)	8 (50.0)	0 (0.0)	3 (50.0)
	**Other**	12 (9.9)	9 (19.6)	1 (2.3)	2 (12.5)	0 (0.0)	0 (0.0)
**T-stage at diagnosis**							
	**1–2**	39 (32.3)	6 (13.0)	16 (36.4)	10 (62.5)	4 (44.4)	3 (50.0)
	**3–4**	56 (46.3)	27 (58.7)	21 (47.7)	4 (25.0)	2 (22.2)	2 (33.3)
	**Tx**	26 (21.5)	13 (28.3)	7 (15.9)	2 (12.5)	3 (33.3)	1 (16.7)
**N-stage at diagnosis**							
	**0**	52 (43.0)	27 (58.7)	8 (18.2)	7 (43.8)	5 (55.6)	5 (83.3)
	**1–3**	44 (36.4)	7 (15.2)	28 (63.6)	7 (43.8)	1 (11.1)	1 (16.7)
	**Nx**	25 (20.7)	12 (26.1)	8 (18.2)	2 (12.5)	3 (33.3)	0 (0.0)
**M-stage at diagnosis**							
	**0**	100 (82.6)	36 (78.3)	39 (88.6)	14 (87.5)	7 (77.8)	4 (66.7)
	**1**	19 (15.7)	8 (17.4)	5 (11.4)	2 (12.5)	2 (22.2)	2 (33.3)
	**Mx**	2 (1.7)	2 (4.3)	0 (0.0)	0 (0.0)	0 (0.0)	0 (0.0)
**Initial treatment intent**							
	**Curative**	99 (83.5)	35 (76.1)	39 (88.6)	14 (87.5)	7 (77.8)	4 (66.7)
	**Palliative**	22 (18.2)	11 (23.9)	5 (11.4)	2 (12.5)	2 (22.2)	2 (33.3)
**R/M disease after initial curative treatment (*n* = 99)**							
	**Yes**	63 (63.4)	28 (80.0)	24 (61.5)	6 (42.9)	4 (57.1)	1 (25%)
	**No**	36 (36.4)	7 (20.0)	15 (38.5)	8 (57.1)	3 (42.9)	3 (75%)
**Underwent surgery primary tumor**							
	**Yes**	101 (83.5)	36 (78.3)	38 (86.4)	15 (93.8)	8 (88.9)	4 (66.7)
	**No**	20 (16.5)	10 (21.7)	6 (13.6)	1 (6.3)	1 (11.1)	2 (33.3)
**Palliative systemic therapy**							
	**Yes**	57 (47.1)	18 (39.1)	28 (63.6)	4 (25.0)	5 (55.6)	2 (33.3)
	**No**	64 (52.9)	28 (60.9)	16 (36.4)	12 (75.0)	4 (44.4)	4 (66.7)
**Lines of systemic therapy**							
	**Median (range)**	1 (1–7)	1 (1–4)	2 (1–5)	2 (1–7)	2 (1–2)	1 (1)
**First line systemic therapy**							
	**Chemotherapy**	18 (14.9)	11 (23.9)	2 (4.5)	2 (12.5)	3 (33.3)	0 (0.0)
	**Targeted**	12 (9.9)	6 (13.0)	1 (2.3)	2 (12.5)	1 (11.1)	2 (33.3)
	**Hormonal**	17 (14.0)	0 (0.0)	16 (36.4)	0 (0.0)	1 (11.1)	0 (0.0)
	**Immunotherapy**	1 (0.8)	0 (0.0)	1 (2.3)	0 (0.0)	0 (0.0)	0 (0.0)
	**Combination**	8 (6.6)	0 (0.0)	8 (18.2)	0 (0.0)	0 (0.0)	0 (0.0)
	**Other**	1 (0.8)	1 (2.2)	0 (0.0)	0 (0.0)	0 (0.0)	0 (0.0)

## Data Availability

NGS data (TSO500) is available in the European Genome-Phenome Archive after reasonable request (Study ID EGAS00001006232).

## Supplementary Materials

The following supporting information can be downloaded at: https://www.mdpi.com/article/10.3390/cancers14174156/s1. Supplementary Table S1: Genes incorporated in the FusionPlex^®^ RadboudV1. Supplementary Table S2: Genes incorporated in TruSight Oncology 500 panel. Supplementary Table S3: virtual TSO500 panel that mainly contains genes in which aberrations may lead to targeted treatment. Supplementary Table S4: Case number, Subtype, Gene fusion 1, Gene fusion 1 detail, Gene fusion 2, Gene fusion 2 detail. Supplementary Figures S1–S4.
