# Supplementary material for: Identification of Fusion Genes and Targets for Genetically Matched Therapies in a Large Cohort of Salivary Gland Cancer Patients
Source: Cancers (Basel). 2022 Aug 27;14(17):4156. doi: 10.3390/cancers14174156 (PMC9454424; doi:10.3390/cancers14174156)
Supplement: Supplementary file 1 [file cancers-14-04156-s001.zip › Supplementary Figure S1-S4.pdf]

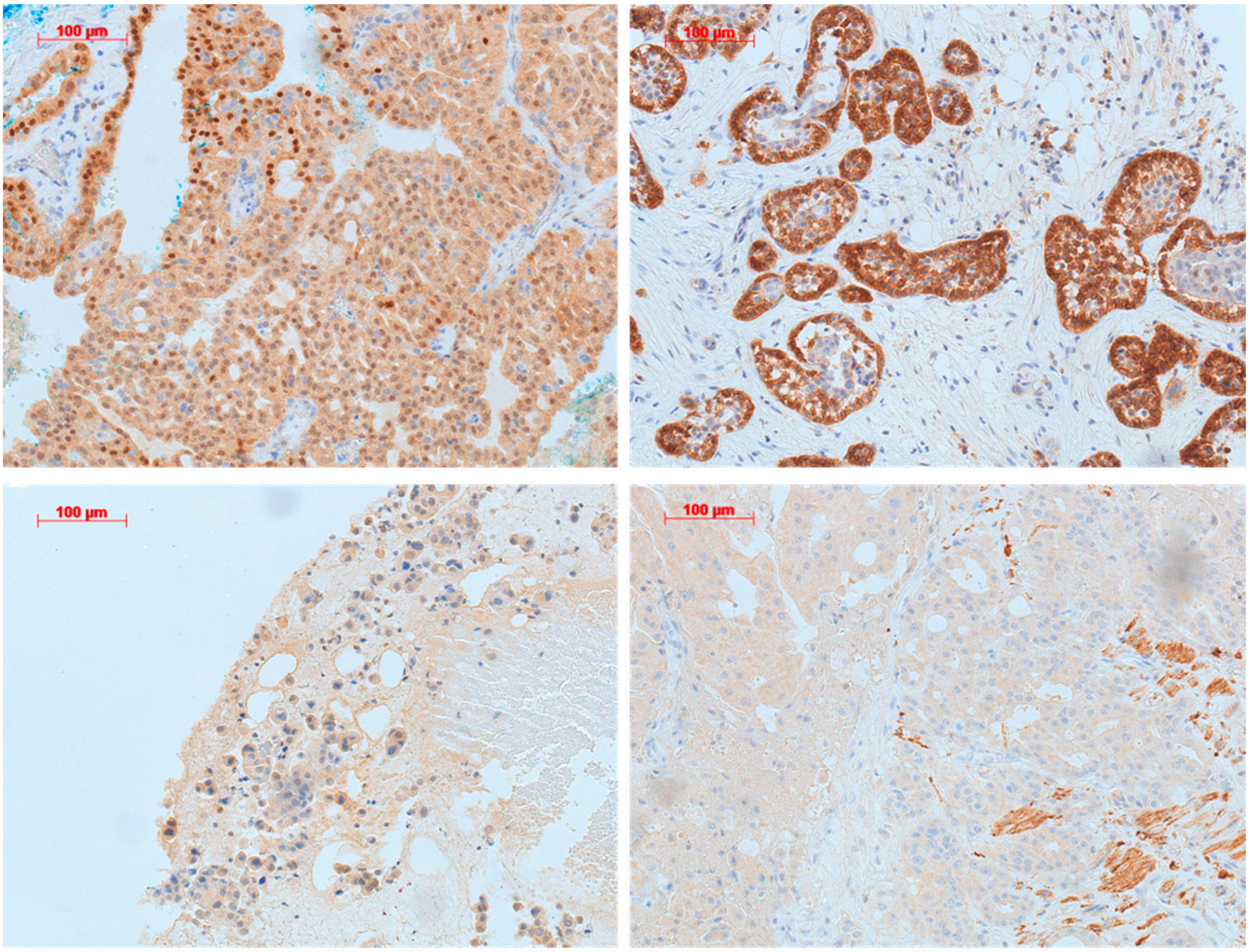

Supplementary Figure S1 Examples of pan-TRK immunohistochemistry (IHC). Top left: secretory carcinoma case harboring an ETV6-NTRK3 gene fusion. Top right: AdCC case scored as positive. Bottom left: SDC case scored as moderate positive. Bottom right: SDC scored as weak/dubious positive.

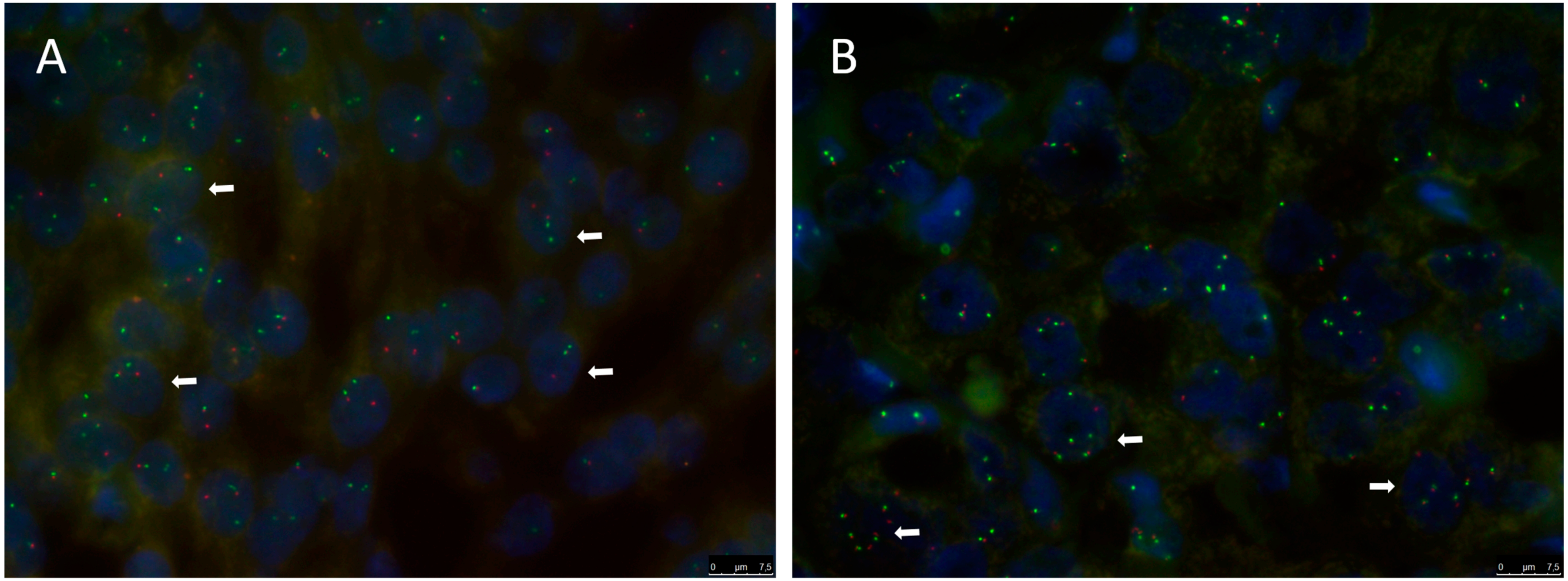

Supplementary Figure S2 Examples of NTRK FISH results. A: NTRK3 FISH in a secretory carcinoma case harboring an ETV6-NTRK3 gene fusion (examples of positive cells indicated with white arrows). B. NTRK1 FISH in a SDC case without harboring a NTRK gene fusion, in which extensive polysomy is detected and FISH was scored as dubious (examples of cells with polysomy indicated with white arrows).

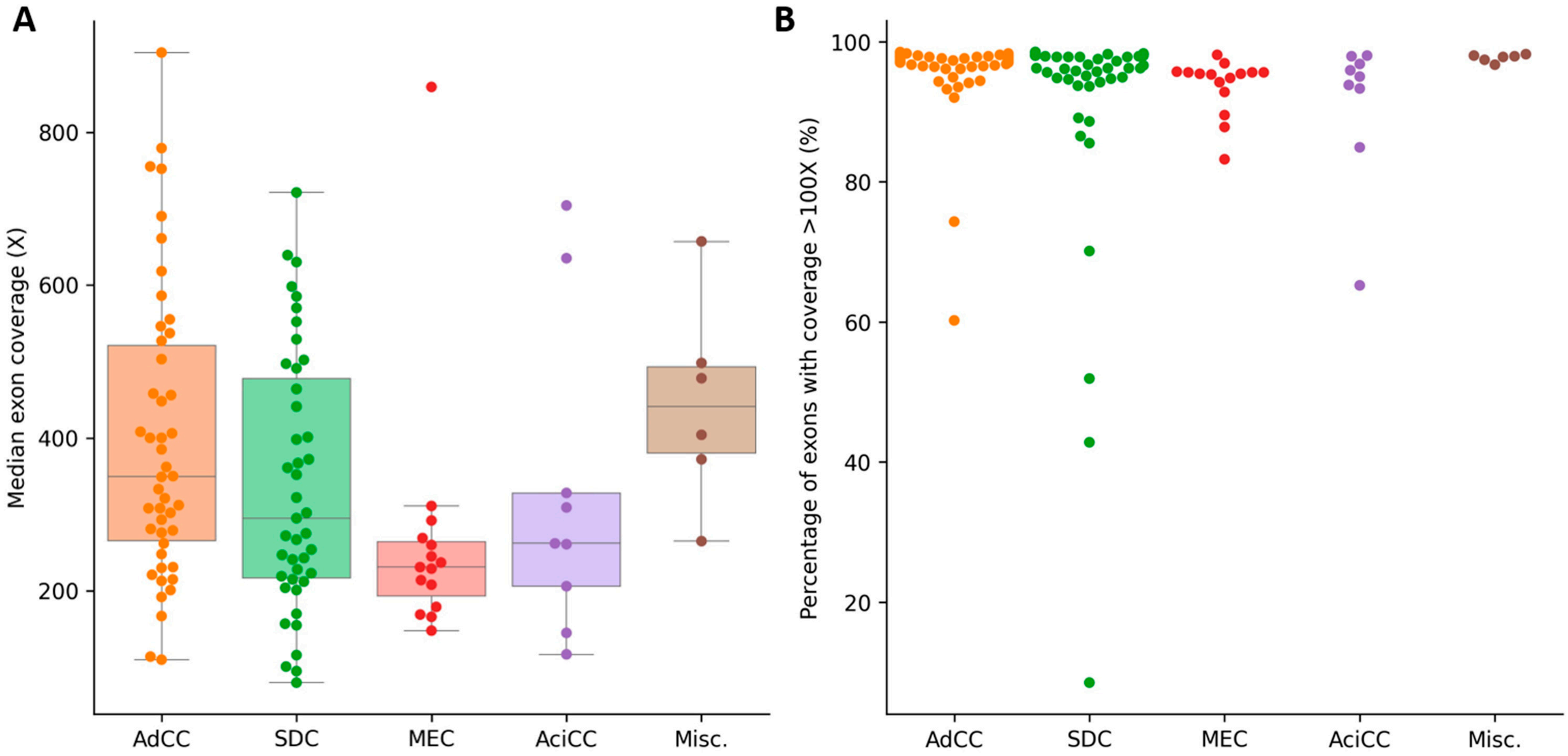

Supplementary Figure S3 Coverage metrics of TSO500 panel. A: median unique exon coverage, sorted per subtype. B: percentage of exons with unique coverage >100X, sorted per subtype. Abbreviations: SDC: salivary duct carcinoma, AdCC: adenoid cystic carcinoma, MEC: mucoepidermoid carcinoma, ACiCC: acinic cell carcinoma, misc.: miscellaneous.

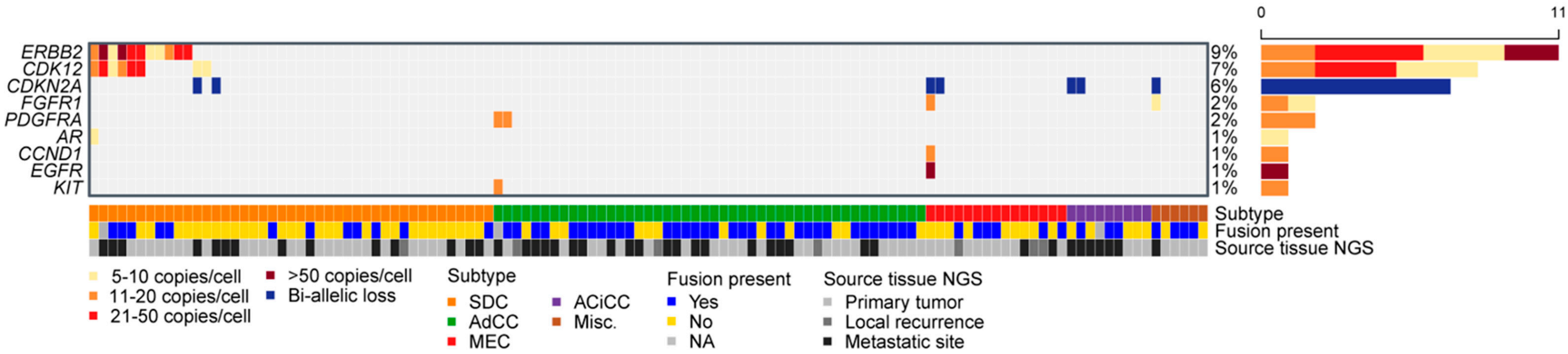

Supplementary Figure S4 Identified copy number variants, sorted for subtypes. Abbreviations: SDC: salivary duct carcinoma, AdCC: adenoid cystic carcinoma, MEC: mucoepidermoid carcinoma, AciCC: acinic cell carcinoma, Misc.: miscellaneous
