# Supplementary material for: Identification of Fusion Genes and Targets for Genetically Matched Therapies in a Large Cohort of Salivary Gland Cancer Patients
Source: Cancers (Basel). 2022 Aug 27;14(17):4156. doi: 10.3390/cancers14174156 (PMC9454424; doi:10.3390/cancers14174156)
Supplement: Supplementary file 1 [file cancers-14-04156-s001.zip › Supplementary Tables S1-S3.pdf]

Supplementary Table S1: Genes incorporated in the FusionPlex® RadboudV1

Gene	NM Reference	Coverage (exons), 5' or 3'directionality not shown
**ABL1**	NM_005157	exon 1, 2, 3, 4
**ABL2**	NM_007314	exon 2, 3, 4, 5, 6
**ALK**	NM_004304	exon 2, 4, 6, 10, 16, 17, 18, 19, 20, 21, 22, 23, 26
**BCOR**	NM_001123385	exon 6, 7, 8, 12, 14, 15
	NM_017745	exon 8
**BRAF**	NM_004333	exon 1, 2, 3, 7, 8, 9, 10, 11, 12, 13, 15, 16
**CAMTA1**	NM_015215	exon 3, 8, 9, 10
**CIC**	NM_015125	exon 18, 19, 20
**EGFR**	NM_005228	exon 1, 7, 8, 9, 16, 19, 20, 24, 25
**ERBB2**	NM_004448	exon 4, 5, 23, 24, 25, 26
**ERG**	NM_004449	exon 2, 3, 4, 5, 6, 7, 8, 9, 10, 11
**ETV6**	NM_001987	exon 1, 2, 3, 4, 5, 6, 7
**EWSR1**	NM_005243	exon 4, 5, 6, 7, 8, 9, 10, 11, 12, 13, 14
**FGFR1**	NM_015850	exon 2, 3, 4, 5, 6, 7, 8, 9, 10, 11, 12, 17
**FGFR2**	NM_000141	exon 2, 5, 7, 8, 9, 10, 16, 17
**FGFR3**	NM_000142	exon 3, 5, 8, 9, 10, 16, 17
**FOS**	NM_005252	exon 1, 2, 3, 4
**FOSB**	NM_006732	exon 1, 2, 3
	NM_001114171	exon 1, 2, 3
**FOXO1**	NM_002015	exon 1, 2, 3
**FUS**	NM_004960	exon 3, 4, 5, 6, 7, 8, 9, 10, 11, 13, 14
**GLI1**	NM_005269	exon 4, 5, 6, 7
**HMGA2**	NM_003483	exon 1, 2, 3, 4, 5
**JAZF1**	NM_175061	exon 2, 3, 4
**MALT1**	NM_006785	exon 2, 3, 4, 5, 6, 7, 9, 10
**MAML2**	NM_032427	exon 2, 3
**MET**	NM_000245	exon 2, 4, 5, 6, 13, 14, 15, 16, 17, 21
**MKL2**	NM_014048	exon 11, 12, 13
**MYB**	NM_001130173	exon 7, 8, 9, 11, 12, 13, 14, 15, 16
**MYBL1**	NM_001080416	exon 6, 7, 8, 9, 10, 11, 12, 13, 14, 15, 16
	NM_001144755	exon 8
**NCOA1**	NM_147223	exon 12, 13, 14, 15
**NCOA2**	NM_006540	exon 11, 12, 13, 14, 15, 16
**NFIB**	NM_005596	exon 5, 6, 7, 8, 9
	NM_001190737	exon 9, 10
**NR4A3**	NM_173200	exon 3, 4
**NR4A3**	NM_006981	exon 4
	NM_013962	exon 1
**NRG1**	NM_004495	exon 1, 2, 3, 6
	NM_013957	exon 1, 4, 8
**NTRK1**	NM_002529	exon 2, 4, 6, 8, 10, 11, 12, 13, 14, 15

**NTRK2**	NM_006180	exon 5, 7, 9, 11, 12, 13, 14, 15, 16, 17
**NTRK3**	NM_002530	exon 4, 7, 10, 12, 13, 14, 15, 16
	NM_001007156	exon 15
**PDGFB**	NM_002608	exon 2, 3
**PDGFRB**	NM_002609	exon 8, 9, 10, 11, 12, 13, 14
**PHF1**	NM_024165	exon 1, 2, 3, 4, 5, 6, 7, 8
**PLAG1**	NM_002655	exon 1, 2, 3, 4
**PPARG**	NM_015869	exon 1, 2, 3
**PRKD1**	NM_002742	exon 7, 8, 9, 10, 11, 12, 13, 14, 15, 16, 17
**PRKD2**	NM_016457	exon 7, 8, 9, 10, 11, 12, 13, 14, 15, 16, 17
**PRKD3**	NM_005813	exon 7, 8, 9, 10, 11, 12, 13, 14, 15, 16, 17
	NM_133509	exon 11
**RAD51B**	NM_002877	exon 11
	NM_133510	exon 1, 2, 3, 4, 5, 6, 7, 8, 9, 10, 11
**RAF1**	NM_002880	exon 4, 5, 6, 7, 8, 9, 10, 11, 12
**RET**	NM_020975	exon 8, 9, 10, 11, 12, 13, 14
	NM_020630	exon 2, 4, 6
**ROS1**	NM_002944	exon 2, 4, 7, 31, 32, 33, 34, 35, 36, 37
**SS18**	NM_001007559	exon 4, 5, 6, 8, 9, 10, 11
**STAT6**	NM_001178078	exon 1, 2, 3, 4, 5, 6, 7, 15, 16, 17, 18, 19, 20
**TFE3**	NM_006521	exon 2, 3, 4, 5, 6, 7, 8, 10
**THADA**	NM_022065	exon 24, 25, 26, 27, 28, 29, 30, 36, 37
**TMPRSS2**	NM_005656	exon 1, 3, 4, 5, 6
	NM_001135099	exon 1, 2
**USP6**	NM_004505	exon 1, 2, 3
**YWHAE**	NM_006761	exon 5

Supplementary Table S2: Genes incorporated in TruSight Oncology 500 panel

TSO500 panel										
ABL1	*BRD4*	*CUX1*	*FAM175A*	*GATA6*	*IGF1*	*MAP3K13*	*NOTCH4*	*POLE*	*RPTOR*	*TAF1*
ABL2	*BRIP1*	*CXCR4*	*FAM46C*	*GEN1*	*IGF1R*	*MAP3K14*	*NPM1*	*PPARG*	*RUNX1*	*TBX3*
ACVR1	*BTG1*	*CYLD*	*FANCA*	*GID4*	*IGF2*	*MAP3K4*	*NRAS*	*PPM1D*	*RUNX1T1*	*TCEB1*
ACVR1B	*BTK*	*DAXX*	*FANCC*	*GLI1*	*IKBKE*	*MAPK1*	*NRG1*	*PPP2R1A*	*RYBP*	*TCF3*
AKT1	*C11orf30*	*DCUN1D1*	*FANCD2*	*GNA11*	*IKZF1*	*MAPK3*	*NSD1*	*PPP2R2A*	*SDHA*	*TCF7L2*
AKT2	*CALR*	*DDR2*	*FANCE*	*GNA13*	*IL10*	*MAX*	*NTRK1*	*PPP6C*	*SDHAF2*	*TERC*
AKT3	*CARD11*	*DDX41*	*FANCF*	*GNAQ*	*IL7R*	*MCL1*	*NTRK2*	*PRDM1*	*SDHB*	*TERT*
ALK	*CASP8*	*DHX15*	*FANCG*	*GNAS*	*INHA*	*MDC1*	*NTRK3*	*PREX2*	*SDHC*	*TET1*
ALOX12B	*CBFB*	*DICER1*	*FANCI*	*GPR124*	*INHBA*	*MDM2*	*NUP93*	*PRKAR1A*	*SDHD*	*TET2*
ANKRD11	*CBL*	*DIS3*	*FANCL*	*GPS2*	*INPP4A*	*MDM4*	*NUTM1*	*PRKCI*	*SETBP1*	*TFE3*
ANKRD26	*CCND1*	*DNAJB1*	*FAS*	*GREM1*	*INPP4B*	*MED12*	*PAK1*	*PRKDC*	*SETD2*	*TFRC*
APC	*CCND2*	*DNMT1*	*FAT1*	*GRIN2A*	*INSR*	*MEF2B*	*PAK3*	*PRSS8*	*SF3B1*	*TGFBR1*
AR	*CCND3*	*DNMT3A*	*FBXW7*	*GRM3*	*IRF2*	*MEN1*	*PAK7*	*PTCH1*	*SH2B3*	*TGFBR2*
ARAF	*CCNE1*	*DNMT3B*	*FGF1*	*GSK3B*	*IRF4*	*MET*	*PALB2*	*PTEN*	*SH2D1A*	*TMEM127*
ARFRP1	*CD274*	*DOT1L*	*FGF10*	*H3F3A*	*IRS1*	*MGA*	*PARK2*	*PTPN11*	*SHQ1*	*TMPRSS2*
ARID1A	*CD276*	*E2F3*	*FGF14*	*H3F3B*	*IRS2*	*MITF*	*PARP1*	*PTPRD*	*SLIT2*	*TNFAIP3*
ARID1B	*CD74*	*EED*	*FGF19*	*H3F3C*	*JAK1*	*MLH1*	*PAX3*	*PTPRS*	*SLX4*	*TNFRSF14*
ARID2	*CD79A*	*EGFL7*	*FGF2*	*HGF*	*JAK2*	*MLL*	*PAX5*	*PTPRT*	*SMAD2*	*TOP1*
ARID5B	*CD79B*	*EGFR*	*FGF23*	*HIST1H1C*	*JAK3*	*MLLT3*	*PAX7*	*QKI*	*SMAD3*	*TOP2A*
ASXL1	*CDC73*	*EIF1AX*	*FGF3*	*HIST1H2BD*	*JUN*	*MPL*	*PAX8*	*RAB35*	*SMAD4*	*TP53*
ASXL2	*CDH1*	*EIF4A2*	*FGF4*	*HIST1H3A*	*KAT6A*	*MRE11A*	*PBRM1*	*RAC1*	*SMARCA4*	*TP63*
ATM	*CDK12*	*EIF4E*	*FGF5*	*HIST1H3B*	*KDM5A*	*MSH2*	*PDCD1*	*RAD21*	*SMARCB1*	*TRAF2*
ATR	*CDK4*	*EML4*	*FGF6*	*HIST1H3C*	*KDM5C*	*MSH3*	*PDCD1LG2*	*RAD50*	*SMARCD1*	*TRAF7*
ATRX	*CDK6*	*EP300*	*FGF7*	*HIST1H3D*	*KDM6A*	*MSH6*	*PDGFRA*	*RAD51*	*SMC1A*	*TSC1*
AURKA	*CDK8*	*EPCAM*	*FGF8*	*HIST1H3E*	*KDR*	*MST1*	*PDGFRB*	*RAD51B*	*SMC3*	*TSC2*
AURKB	*CDKN1A*	*EPHA3*	*FGF9*	*HIST1H3F*	*KEAP1*	*MST1R*	*PDK1*	*RAD51C*	*SMO*	*TSHR*
AXIN1	*CDKN1B*	*EPHA5*	*FGFR1*	*HIST1H3G*	*KEL*	*MTOR*	*PDPK1*	*RAD51D*	*SNCAIP*	*U2AF1*
AXIN2	*CDKN2A*	*EPHA7*	*FGFR2*	*HIST1H3H*	*KIF5B*	*MUTYH*	*PGR*	*RAD52*	*SOCS1*	*VEGFA*
AXL	*CDKN2B*	*EPHB1*	*FGFR3*	*HIST1H3I*	*KIT*	*MYB*	*PHF6*	*RAD54L*	*SOX10*	*VHL*
B2M	*CDKN2C*	*ERBB2*	*FGFR4*	*HIST1H3J*	*KLF4*	*MYC*	*PHOX2B*	*RAF1*	*SOX17*	*VTCN1*
BAP1	*CEBPA*	*ERBB3*	*FH*	*HIST2H3A*	*KLHL6*	*MYCL1*	*PIK3C2B*	*RANBP2*	*SOX2*	*WISP3*
BARD1	*CENPA*	*ERBB4*	*FLCN*	*HIST2H3C*	*KMT2B*	*MYCN*	*PIK3C2G*	*RARA*	*SOX9*	*WT1*
BBC3	*CHD2*	*ERCC1*	*FLI1*	*HIST2H3D*	*KMT2C*	*MYD88*	*PIK3C3*	*RASA1*	*SPEN*	*XIAP*
BCL10	*CHD4*	*ERCC2*	*FLT1*	*HIST3H3*	*KMT2D*	*MYOD1*	*PIK3CA*	*RB1*	*SPOP*	*XPO1*
BCL2	*CHEK1*	*ERCC3*	*FLT3*	*HLA-A*	*KRAS*	*NAB2*	*PIK3CB*	*RBM10*	*SPTA1*	*XRCC2*
BCL2L1	*CHEK2*	*ERCC4*	*FLT4*	*HLA-B*	*LAMP1*	*NBN*	*PIK3CD*	*RECQL4*	*SRC*	*YAP1*
BCL2L11	*CIC*	*ERCC5*	*FOXA1*	*HLA-C*	*LATS1*	*NCOA3*	*PIK3CG*	*REL*	*SRSF2*	*YES1*
BCL2L2	*CREBBP*	*ERG*	*FOXL2*	*HNF1A*	*LATS2*	*NCOR1*	*PIK3R1*	*RET*	*STAG1*	*ZBTB2*
BCL6	*CRKL*	*ERRFI1*	*FOXO1*	*HNRNPK*	*LMO1*	*NEGR1*	*PIK3R2*	*RFWD2*	*STAG2*	*ZBTB7A*
BCOR	*CRLF2*	*ESR1*	*FOXP1*	*HOXB13*	*LRP1B*	*NF1*	*PIK3R3*	*RHEB*	*STAT3*	*ZFHX3*
BCORL1	*CSF1R*	*ETS1*	*FRS2*	*HRAS*	*LYN*	*NF2*	*PIM1*	*RHOA*	*STAT4*	*ZNF217*
BCR	*CSF3R*	*ETV1*	*FUBP1*	*HSD3B1*	*LZTR1*	*NFE2L2*	*PLCG2*	*RICTOR*	*STAT5A*	*ZNF703*
BIRC3	*CSNK1A1*	*ETV4*	*FYN*	*HSP90AA1*	*MAGI2*	*NFKBIA*	*PLK2*	*RIT1*	*STAT5B*	*ZRSR2*

BLM	*CTCF*	*ETV5*	*GABRA6*	*ICOSLG*	*MALT1*	*NKX2-1*	*PMAIP1*	*RNF43*	*STK11*
BMPR1A	*CTLA4*	*ETV6*	*GATA1*	*ID3*	*MAP2K1*	*NKX3-1*	*PMS1*	*ROS1*	*STK40*
BRAF	*CTNNA1*	*EWSR1*	*GATA2*	*IDH1*	*MAP2K2*	*NOTCH1*	*PMS2*	*RPS6KA4*	*SUFU*
BRCA1	*CTNNB1*	*EZH2*	*GATA3*	*IDH2*	*MAP2K4*	*NOTCH2*	*PNRC1*	*RPS6KB1*	*SUZ12*
BRCA2	*CUL3*	*FAM123B*	*GATA4*	*IFNGR1*	*MAP3K1*	*NOTCH3*	*POLD1*	*RPS6KB2*	*SYK*

Supplementary Table S3: virtual TSO500 panel that mainly contains genes in which aberrations may lead to targeted treatment

Analyzed genes	TS/O/R[1]	Counted as targetable[2]
AR	O,R	No
AKT1	O	Mut/Amp
AKT2	O	Mut/Amp
AKT3	O	Mut/Amp
ALK	O,R	Fus[3]
ATM	TS	Bi-allelic inactivation
BARD1	TS	Bi-allelic inactivation
BRAF	O	Mut/Fus[3]
BRCA1	TS	Bi-allelic inactivation
BRCA2	TS	Bi-allelic inactivation
BRIP1	TS	Bi-allelic inactivation
B2M	R	No
CCND1	O	Amp
CDK12	TS,O	Bi-allelic inactivation
CDK4	O	Mut/Amp
CDK6	O	Amp
CDKN2A	TS	Bi-allelic inactivation
EGFR	O,R	Mut/Fus[3]
ERBB2	O	Mut/Amp
ERBB4	O	Mut
FANCL	TS	Bi-allelic inactivation
FGFR1	O	Mut/Amp/Fus[3]
FGFR2	O	Mut/Amp/Fus[3]
FGFR3	O	Mut/Amp/Fus[3]
FGFR4	O	Mut/Amp
HRAS	O	Mut
JAK1	R	No
JAK2	O,R	No
KIT	O,R	Mut/Amp
KRAS	O	Mut (only G12C)
MAP2K1	O	Mut
MAP2K2	O	Mut
MAP2K4	TS	Bi-allelic inactivation
MAP3K1	TS	Bi-allelic inactivation
MET	O	Mut/Amp/Fus[3]
MLH1	TS	Bi-allelic inactivation
MSH2	TS	Bi-allelic inactivation
MSH6	TS	Bi-allelic inactivation
NOTCH1	TS, O	Activating Mut
NOTCH2	O	Activating Mut
NOTCH3	O	Activating Mut

NOTCH4	TS, O	No
NRAS	O	Mut
PALB2	TS	Bi-allelic inactivation
PDGFRA	O	Mut/Amp
PDGFRB	O	Mut/Amp
PIK3CA	O	Mut
PIK3R1	TS	Bi-allelic inactivation/activating mutations
PIK3R2	TS	Bi-allelic inactivation
POLE	TS	No
PPP2R2A	TS	Bi-allelic inactivation
PTEN	TS	Bi-allelic inactivation
RAD51B	TS	Bi-allelic inactivation
RAD51C	TS	Bi-allelic inactivation
RAD51D	TS	Bi-allelic inactivation
RAD54L	TS	Bi-allelic inactivation
RAF1	O	No
RET	O	Mut/Fus[3]
TP53	TS	No
TSC1	TS	Bi-allelic inactivation
TSC2	TS	Bi-allelic inactivation
MSI	-	≥25% unstable sites; in case of 10-25% unstable sites, confirm with MMR IHC
TMB	-	≥15mut/Mb

1. TS: Tumor suppressor; O: Oncogene; R: Resistance gene
2. Only (likely) pathogenic mutations were counted as targetable, scored following recommendations of the American College of Medical Genetics and Genomics and the Association for Molecular Pathology. Bi-allelic inactivation: mut and/or loss.
Abbreviations: Mut: mutation; Amp: amplification; Fus: fusion; MSI: microsatellite instability; MMR: mismatch repair; IHC: immunohistochemistry; TMB: tumor mutational burden.
3. Fusions are not detectable by the TSO500 panel, but fusions in these genes were detectable by the FusionPlex® RadboudV1 panel that was used too in this study. In this panel fusions in these genes were counted as actionable: *ABL1, ALK, BRAF, EGFR, FGFR1-3, MAML2 , MET, NRG1, NTRK1-3, RET* and *ROS1.*
